# The Curious History of L'Hotel-Dieu De Paris
*The two previous articles appeared on March 8 and 29.


**Published:** 1913-04-12

**Authors:** 


### THE CURIOUS HISTORY OF L'HOTEL-DIEU DE PARIS.*

### III.—ITS CONNECTION WITH MEDICAL TEACHING.

The outcome of Christian charity and the offshoot of the church itself, the Hôtel-Dieu took root and prospered in the shadow of Notre Dame. In 1506, when its administration was secularised, it established colonies in other parts of the city, the hospitals La Charité and Saint-Louis. Although on the breaking of the tie that bound it to the old church it might have quitted the city, the habit of centuries asserted itself and it remained where it had always been. Not far away, in the Rue des Ecoliers, since renamed Rue du Fouarre, there arose in the thirteenth century the Faculty of Medicine, which in the fourteenth was transferred to the Rue de la Bûcherie. It remained here until the Revolution, when it was rebuilt on the site it now occupies.

### Bachelor Apprentices.

Before this second removal teaching in the Faculty was mainly theoretical, but practical instruction was not unknown. Clinical teaching was of three kinds, that given in the Faculty, that given in private by physicians, and that given in hospital. Free consultations were given at the Faculty every Saturday from 10 a.m. to noon, in which six doctors, three senior and three junior, chosen by rota, took part. Bachelors of medicine attended also, while the masters examined the patients, explained their maladies in Latin to the students, and dictated prescriptions. The date is unknown at which these free consultations were first started, but the text of a notice still exists which the Faculty caused to be placed on the walls of the city in 1637 announcing that they would be given. It is probable, however, that they occurred before this. It is also not clear as to when originated the custom of apprenticing bachelors to doctors, but it is shown that in the seventeenth and eighteenth centuries doctors of repute were accompanied on their visits and consultations by bachelors of medicine who lodged with and were taught by them.

The doctors ambled through the town, first on mules and later on horseback, followed by pupils, who were, when the doctors made visits, able to perfect themselves in the art of questioning patients, drawing up prescriptions and obtaining the necessary explanations. On occasions Latin served as the medium for exchanging confidences between master and pupil. A grand consultation was the occasion for the assembling of many doctors, each accompanied by his pupils. The latter were the witnesses of the courteous, but subtle and impassioned, controversy to which the case gave rise. At times also, on the invitation of the *Dean of the Consultation*, who took the part of president, the pupils were invited to express an opinion. When they had gained their master's confidence, they were allowed to treat on their own responsibility some of the less serious cases at the hospital. Clinical teaching took place during the morning visits. It has been stated that medical students, or *philiatres* as they were then called, only succeeded in gaining admission to the hospital at the end of the seventeenth century, but it is very probable that they had obtained this privilege at a much earlier period. Be this as it may, an edict of 1707 made it obligatory on students to take out a course of two years' study at the Hôtel-Dieu, and it was forbidden for more than five students to take service under the same chief. This hospital was the most popular with students at that time because of its proximity to the Faculty and the Quartier Latin.

### A Morning Visit to the Hospital.

It is interesting to picture the scene of a morning visit to the hospital. About 8 a.m. the physician arrives at the hospital, and having descended from his mule under the porch of the entrance, proceeds into the chapel or Salle Saint-Thomas, where surgeon-companions, bachelors and *philiatres* await him. He joins the *topique* or surgeon-companion on duty in the wards, whose business it is to collect the prescriptions given by the master (at a later date his importance was still further enhanced by his being given a portfolio in which to place these, a custom which still persists), and at once the visit begins according to the customary rite. The "chief" goes from bed to bed, stopping only for a moment or two at each, questions the patient, the *topique* and the *cheftaine*, or head nurse, examines the fæces, the tongue, the pulse and the urine, discourses a little in Latin, and finally prescribes. Diet, purgation, the enema and bleeding play the chief part in his therapeutic armament. Bachelors and *philiatres*, to lose nothing of his flow of wisdom, crush round him, and their example is followed by the young and zealous surgeon-companions. Collisions and imprecations ensue, which an old-standing rivalry does but make the more bitter. That such was actually the case is proved by the demand made by the students in 1727, who, relying on the edict of 1707, claimed the right to be in the front rank when a medical visit was in progress, a right which the surgeon companions with equal vehemence claimed as their own. Clinical teaching, in the proper sense of the word, was, however, almost entirely lacking, partly on account of the large number of patients, partly by the close proximity of the beds, which rendered a proper examination extremely difficult if not entirely impossible, and partly on account of the lack of methodical instruction undertaken by competent and responsible teachers.

### The Pioneer of Clinical Teaching.

This was felt by the old Faculty, and in 1775 two *doctor-regents*, Duchanoy and Jumelin, pub-

* The two previous articles appeared on March 8 and 29.

lished a memoir showing *the utility of creating a clinical school of medicine*, but the Revolution interfered with this project, for in 1792 all teaching bodies, including the old Faculty, were suppressed. The credit of being the pioneer of clinical teaching in France must, however, be given to Desbois de Rochefort, who, born in Paris in 1750, became physician to the Charité Hospital at the age of thirty, and immediately started a course of clinical lectures at the bedside which had a tremendous vogue. The Hôtel-Dieu was deserted, for all rushed to the Charité to hear the new master. His career was unfortunately short-lived, for he died of phthisis at the age of thirty-six. His only work, a treatise on materia medica and the art of prescribing, which has served as a model for all works of a similar kind since his time, was published after the master's death by Corvisart, one of his pupils and afterwards physician to the same hospital. In 1794 the Convention re-established the teaching bodies, and the Parisian Faculty of Medicine was reopened under the name of "Ecole Centrale de Santé." The new scheme was fathered by Fourcroy at the instigation of Chaussier, and he insisted on a change in the methods of administration of the Faculty as well as on the teaching of medicine from a practical standpoint. Twelve professors were appointed, with twelve assistant professors, of whom three were to teach clinical medicine, one in the wards of the Charité, one in the out-patient department of the Hôtel-Dieu and the third in the *clinique d'exception*, where rare and complicated cases were dealt with. In 1823 an edict was promulgated creating four chairs of clinical medicine, and from that date the Hôtel-Dieu possessed a teacher of clinical medicine in the wards as well as in the out-patient department. The list of famous men connected with the hospital is a long one and included Récamier, Chomel, Trousseau, Grisolle, Béhier Sée Dieulafoy, Rostan, Piorry and Gilbert. The hospital has during its long life seen many changes in medical treatment as well as the rise in importance of the art of surgery. But it still ministers to the wants of ailing humanity, and so continues to fulfil the objects its pious founders had in view when they built the original Hôtel-Dieu.

